# Primary Mediastinal Ewing’s Sarcoma: Post Hoc Analysis from Two International Multicenter Prospective Randomized Trials

**DOI:** 10.3390/cancers17010118

**Published:** 2025-01-02

**Authors:** Theresa Stork, Andreas Ranft, Clemens Aigner, Heribert Jurgens, Ruth L. Ladenstein, Beate Timmermann, Henk Van den Berg, Uta Dirksen, Stéphane Collaud

**Affiliations:** 1 Department of Thoracic Surgery, Ruhrlandklinik, University of Duisburg-Essen, 45239 Essen, Germany; storkt@kliniken-koeln.de (T.S.);; 2 Department of Thoracic Surgery, Cologne-Merheim Hospital, University of Witten/Herdecke, 51109 Cologne, Germany; 3 German Cancer Consortium (DKTK), Center Essen, West German Cancer Center, 45147 Essen, Germany; 4 Department of Pediatrics III, University Hospital Essen, University of Duisburg-Essen, 45147 Essen, Germany; 5 Department of Thoracic Surgery, Medical University of Vienna, 1090 Vienna, Austria; 6 Pediatric Oncology and Hematology, University Hospital Muenster, 48149 Muenster, Germany; h.jurgens@uni-muenster.de; 7 St Anna Children’s Hospital and Children’s Cancer Research Institute, Department of Studies and Statistics for Integrated Research and Projects, Medical University of Vienna, 1090 Vienna, Austria; ruth.ladenstein@ccri.at; 8 West German Center for Proton Therapy, University Hospital Essen, University of Duisburg-Essen, 45147 Essen, Germany; 9 Department of Paediatrics, Academisch Medisch Centrum Universiteit van Amsterdam, 1105 Amsterdam, The Netherlands

**Keywords:** mediastinal, Ewing sarcoma, soft tissue sarcoma, sarcoma, multimodal therapy

## Abstract

Objective: Ewing sarcoma (EWS) of the mediastinum is extremely rare, with only a few cases reported in the literature. We aimed to gain a better understanding of primary mediastinal EWS, describing patients treated within two international, multicenter, prospective, randomized EWS trials. Methods: Data from patients with primary mediastinal EWS were retrieved from the database of the EURO-E.W.I.N.G.99 (ClinicalTrials.gov identifier: NCT00020566) and EWING 2008 (ClinicalTrials.gov identifier: NCT00987636) trials. Patient and treatment characteristics were analyzed. Results: Out of 2969 patients with EWS, 9 (0.3%) had primary mediastinal EWS. The median age at diagnosis was 30.5 years (4 to 49). At the time of diagnosis, *n* = 3 (33%) patients had synchronous metastases. All patients underwent multiagent chemotherapy. Local therapy for non-metastatic patients was surgery (*n* = 2, 22%), surgery and radiotherapy (*n* = 2, 22%) or radiotherapy alone (*n* = 2, 22%). Surgery consisted of extended resections in most patients (*n* = 3, 33%). Five-year survival for the whole cohort was 64%. Apart from one patient who was lost to follow-up, all patients (*n* = 4) who had undergone surgery were alive at the end of follow-up. Conclusions: Primary mediastinal ES is extremely rare, with a prevalence of 0.3% among EWS. Five-year survival was favorable compared to primary mediastinal sarcoma of all histologies and in line with EWS of different origin.

## Simple Summary

Ewing sarcomas are rare, aggressive tumors, occurring mostly in children and adolescents. They usually derive from the bone; however, extraskeletal Ewing sarcomas develop in the soft tissue. Ewing sarcoma of the mediastinum is extremely rare; there are only a few cases reported in the literature. The outcome is usually poor with about 30% 5-year survival. We retrieved data from two international, multicenter, prospective, clinical trials investigating the optimal treatment for patients with Ewing sarcoma. We then analyzed the patient and treatment characteristics to gain a better understanding of this rare tumor entity. This study is the first to describe the prevalence of mediastinal Ewing sarcoma in a homogeneously treated patient cohort.

## 1. Introduction

Ewing sarcomas (EWS) represent a highly aggressive, heterogenous group of small, blue, round cell tumors. EWS predominantly occurs in children, adolescents and young adults with a peak of incidence during the second decade of life. These tumors most commonly derive from the osseous structures of the pelvis, axial skeleton and femur. However, they can also develop in soft tissue [1]. Extraskeletal Ewing sarcomas (EES) occur with an incidence of about 0.4/1,000,000. Notably, patients with EES tend to be older than those with EWS of the bone [2]. The majority of cases occur in patients younger than 5 or older than 35 years [3]. Mediastinal soft tissue sarcomas (STS) represent about 1% of all soft tissue sarcomas [4,5]. EWS of the mediastinum is extremely rare, with only a limited number of cases reported in the literature [6,7,8]. A multidisciplinary approach is imperative in treating patients with EWS. Since the use of chemotherapy was introduced, the survival rates for patients with localized EWS has improved drastically, rising from about 10% to around 75% [9]. Sadly, patients with metastatic disease at the time of diagnosis—occurring in 20–25% of cases—continue to have poor prognosis [10,11]. The reported survival rates for mediastinal sarcoma are poor, with a 5-year overall survival of about 30% [5]. The standard treatment for EWS consists of a multimodal approach that combines multiagent chemotherapy, surgery and/or radiation therapy [12,13,14]. Chemotherapy regimens include vincristine, doxorubicin, cyclophosphamide, ifosfamide, actinomycin and etoposide. Although the same drugs are commonly used, the exact regimens differ between Europe and North America [14].

The primary goal of surgery is the complete resection of the tumor with free margins (R0 resection). Patients with suspected EWS should be referred to specialized surgeons at experienced centers even before diagnosis. Lower local recurrence rates have been reported when both tumor biopsy and tumor resection were performed at the same specialized center [15]. Patients with EWS do not benefit from tumor debulking [16]. If complete resections cannot be achieved, definitive radiotherapy should be performed. Due to their localization with close anatomical contact to unresectable structures like the heart, complete resection can be especially difficult to achieve in mediastinal sarcomas.

In this study, we aimed to gain a better understanding of primary mediastinal EWS in describing the patient characteristics and treatment outcomes of patients treated within two international, multicenter, prospective, randomized trials focusing on EWS [13,15,17,18].

## 2. Materials and Methods

All patients were treated within the Euro-E.W.I.N.G.99 (ClinicalTrials.gov identifier: NCT00020566) or EWING 2008 clinical trials (ClinicalTrials.gov identifier: NCT00987636). Both international, multicenter, randomized prospective trials recruited all patients with newly diagnosed EWS and were designed to optimize therapy and survival for patients with EWS.

Patient data were prospectively collected in the database of the Cooperative Ewing Sarcoma Study (CESS) group of the German Society of Pediatric Hematology and Oncology. For the purpose of the current analysis, a systematic search of the database was performed to identify all patients diagnosed with primary mediastinal EWS. Available CT scans were independently reviewed by two experienced surgeons (S.C. and T.S.) to ensure accurate assessment of tumor localization in the mediastinal compartments as described by the International Thymic Malignancy Interest Group (ITMIG) classification [19]. The decision regarding local treatment was performed on a case-by-case basis at the weekly international interdisciplinary tumor board (ITB) of the Cooperative CESS group [20]. In all patients, definitive diagnosis of EWS was made, or reviewed, at a sarcoma reference center with conventional histology, immunohistochemistry and molecular pathology. Surgery was the local therapy of choice in all patients, if tumors were deemed resectable. Patients with tumors classified as unresectable received definitive radiotherapy as their primary local treatment. Additionally, postoperative radiotherapy was performed in patients who underwent incomplete resection, had near tumor resection margins or poor histological response to induction chemotherapy. All patients were treated with a multiagent chemotherapy regimen according to the trial protocols. This included induction chemotherapy with vincristine, ifosfamide, doxorubicin, etoposide (VIDE) followed by risk-stratified consolidation therapy subsequent to local treatment [1,13,14,17,21].

Data were analyzed using GraphPad Prism 5 and IBM SPSS 26 statistical software (IBM Corp., Armonk, NY, USA). Survival probability among patients was analyzed using Kaplan–Meier curves. The impact on survival of the following clinical variables, age, gender, tumor size, surgery, timing of metastases, was assessed in univariate analysis by using log-rank test. A *p*-value ≤ 0.05 was considered statistically significant.

## 3. Results

**Figure 1:**
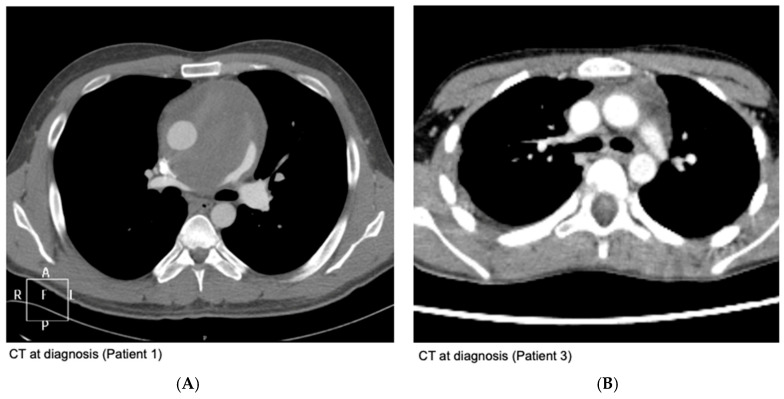
Depicts the variability in appearance of Ewing sarcoma on CT imaging. (**A**) Shows an unresectable tumor located in the middle mediastinum and diffusely infiltrating the heart. (**B**) Shows a small, localized tumor of the anterior mediastinum.

Out of a total of 2969 patients diagnosed with EWS, 9 (0.3%) were identified as having primary mediastinal EWS. A detailed summary of the patient and tumor characteristics is provided in Table 1. The available CT scans at diagnosis of two patients are provided in Figure 1.

**Figure 2:**
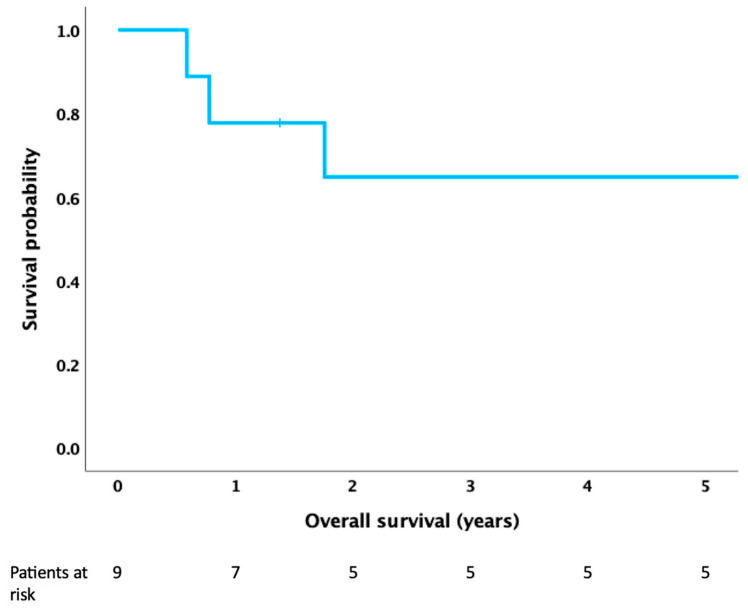
Overall survival for patients with primary mediastinal Ewing sarcoma.

The median age at diagnosis was 30.5 years (range 4 to 49 years). The median tumor size was 12.5 cm (range 6–16 cm). Eight (89%) patients were diagnosed with EWS prior to multimodal treatment. One (11%) patient had upfront surgery, during which an intraoperative frozen section analysis suggested Morbus Castleman. Final histologic examination excluded Morbus Castleman and revealed the definitive diagnosis of EWS. At the time of diagnosis, three patients had synchronous metastases to the lung and pericardium (*n* = 1, 11%), bone (*n* = 1, 11%) or liver and bone (*n* = 1, 11%). Six patients presented with pleural effusion at the time of diagnosis. A pleural puncture with cytologic assessment was performed in five patients. Malignant pleural effusion was identified in two of these patients. All patients in the cohort underwent multiagent chemotherapy, consisting of vincristine, ifosfamide, doxorubicin, etoposide (VIDE) and vincristine, actinomycin D, ifosfamide (VAI) in most patients (*n* = 5, 55%). Local therapy for non-metastatic primary mediastinal EWS was surgery alone (*n* = 2, 22%), a combination of surgery and radiotherapy (*n* = 2, 22%) and radiotherapy alone (*n* = 2, 22%). Surgery consisted of extended resections in most patients (*n* = 3, 75%). Extended resections included the resection of lung parenchyma, pericardium, esophageal muscle layer, atrium and diaphragm. The median follow-up was 5.6 years (range 0.6–9.6 years). The 5-year progression-free survival was 60%. The overall 5-year survival for the whole patient cohort was 64% (Figure 2). Two patients who presented with synchronous metastases died of disease after progressive disease while being treated with VIDE chemotherapy within the first year after diagnosis. One patient with synchronous osseous metastases was treated with high-dose busulfan and melphalan (BuMel) after progression and died of disease 1.8 years following the diagnosis. Apart from one patient who was lost to follow-up, all patients who underwent surgery were alive at the end of the follow-up period. Patients with metastases at the time of diagnosis had a significantly lower overall (100% vs. 0%, *p* = 0.002; HR = 13 (95% CI 1.056–160.1)) and progression-free 5-year survival (100% vs. 0%, *p* = 0.006; HR = 10.94 (95% CI 1.008–118.8)) when compared to patients with localized disease. Other clinical factors such as patient age, gender and tumor size did not impact overall nor progression-free survival in the univariate analysis.

## 4. Discussion

Primary mediastinal Ewing sarcoma is an extremely rare tumor entity. Due to its rarity, not much is known regarding patient outcomes and optimal treatment strategy. This is the first analysis on the prevalence and outcome of a homogeneously treated group of patients with primary mediastinal EWS. The retrospective analysis was performed in a large cohort of unselected patients who were registered into two consecutive national and international trials of the Cooperative Ewing Sarcoma Study group (CESS). Data from these patients were collected prospectively. We analyzed patients treated within these two consecutive clinical trials, which used similar chemotherapeutic agents within their study protocols, and which were conducted within a timeframe of less than 10 years. This setting was chosen to minimize the potential confounding effects of major variations in the quality of imaging and therapeutic concepts.

Mediastinal sarcomas are rare, accounting for only 1–2% of soft tissue sarcomas [22]. In the present cohort, based on two prospective, randomized controlled trials investigating newly diagnosed EWS, primary mediastinal origin was found in 0.3% of cases.

The outcome of mediastinal sarcomas is generally considered to be poor as most patients present with already metastatic or locally extensive disease at the time of diagnosis [23,24]. In this cohort, 5-year survival was 64%. Direct comparison with the literature is difficult since no overall survival is published for primary mediastinal EWS. However, survival for primary mediastinal sarcoma in studies investigating all histologic subtypes has been poor with 5-year survival ranging from about 15% in a large cohort of 976 patients [23] to around 36% [25]. This difference compared to our cohort may be due to the young age, the generally good response of EWS to chemotherapy and radiotherapy and the fact that all patients did benefit from central case discussion within a highly specialized dedicated tumor board (ITB of CESS) and from being treated within a standardized clinical trial protocol [26]. The positive impact on the outcome of a dedicated reference tumor board was described elsewhere for patients with metastatic EWS. Patients who had been discussed by the tumor board and whose recommendations were followed had a significantly better overall survival compared to those who did not receive a treatment recommendation (*p* = 0.026) [20]. The good outcome in this cohort might also be explained by the high rates of R0 resection compared to the literature.

Complete tumor resection plays a central role in the treatment of sarcomas. EWS are highly aggressive tumors, infiltrating neighboring structures that then also need to be resected en bloc to obtain complete resection. However, in mediastinal tumors, with their unique localization with surrounding unresectable structures such as the heart and great vessels, R0 resection may be especially difficult to obtain. Imaginative and unconventional resection strategies up to heart explantation and autotransplantation are described in the literature [12,27]. In the largest series about primary mediastinal sarcomas including 976 patients from the National Cancer Database, less than 50% of patients underwent resection. Instead, most patients were treated with radiotherapy and/or chemotherapy. If surgery was performed, a microscopic complete resection could only be achieved in 33.8% of cases [23]. In the present cohort, 44% of patients underwent resection, with an R0 rate of 75%. This is an exceptionally high number for mediastinal sarcoma, especially since all patients needed extended resections to obtain negative margins. This might be due to the fact that all patients were operated on in a specialized center. Another reason could also be the good response of EWS to chemotherapy compared to other mediastinal sarcomas. Unfortunately, data on pathological responses to chemotherapy were not available in all patients. All patients—except one who was lost to follow-up—who underwent surgery were alive at the end of follow-up. This highlights the fact that surgery, even in locally extensive disease, can be curative and should always be considered if the tumor is deemed resectable on (post-induction) preoperative imaging. Full preoperative workup is crucial to identify patients who can undergo complete tumor resection. This may include transesophageal echo, cardiac CT or MRI to identify infiltration of the heart. Pleural effusion is often automatically considered malignant, if it occurs in patients with a known malignancy. However, not all patients who present with pleural effusion have metastatic disease. In our cohort, out of six patients who presented with pleural effusion, five had cytological fluid examination and only two were positive for Ewing sarcomatosis. Since prognosis and treatment are very different in cases of pleural metastases, pleural effusion should always be investigated including cytology and video- assisted thoracoscopic pleural biopsy if required before assuming malignancy.

When mediastinal sarcoma is suspected, an interdisciplinary discussion including a thoracic surgeon with expertise in sarcoma is mandatory to obtain the best treatment strategy. Referral to a sarcoma center prior to tumor diagnosis and biopsy might be practically difficult but can be beneficial to patients. Indeed, patients had a lower risk of local recurrence (14% vs. 32% at 5 years, *p* = 0.035) if biopsy and tumor resection were carried out at the same (specialized) center as reported by a study investigating the risk factors for local recurrence in pelvic Ewing sarcoma [15]. Additionally, an inadequate biopsy or excision may jeopardize further complete tumor resection due to the risk of tumor seeding [28]. The need for larger resections or even amputations in patients with extremity sarcomas has been reported if tumor biopsies have not been carried out appropriately [29].

Patients with EWS do not benefit from tumor debulking or second-look procedures in case of incomplete resection during the first surgery. EWS have been known to be radiosensitive tumors since they were first described. In patients with tumors deemed unresectable or when complete tumor resection would result in mutilation or is associated with an exceptionally high complication rate, definitive radiotherapy should be performed [16]. Radiotherapy can also be considered preoperatively in marginally resectable primary mediastinal EWS, as in some pelvic EWS. Postoperative radiotherapy is always indicated in case of intralesional or marginal resection (R2 or R1 resections). In Europe, radiotherapy is also recommended in patients with a poor histological response and more than 10% of viable tumor cells after induction chemotherapy, regardless of R status [30,31]. Patients with a higher tumor volume (>200 mL) might also benefit from additional radiotherapy after surgical resection [32]. In our cohort, RT was used for definitive and adjuvant purposes. Unfortunately, details of target volumes and doses were not consistently available, and a quality assurance program was not part of the studies. Therefore, we cannot analyze the effectiveness of the applied RT for the patients with mediastinal EWS in our cohort. However, in the upcoming IEuroEwing trial, a retrospective quality assurance program in addition to dose escalation strategies will be established, allowing evaluation of the role of RT.

The presence of metastases at the time of diagnosis is the most important adverse prognostic factor in EWS. Five-year survival in all patients with metastatic disease is about 30%. Patients with lung metastases only tend to have a better prognosis compared to those with primary bone metastases. Patients with more than one bone metastasis show a significantly worse outcome [11]. Patients with disseminated disease might also profit from local therapy of all tumor sites with significantly better event-free survival [11]. In this cohort, all patients with metastases at diagnosis already had extensive metastatic disease including multiple bone metastases and liver metastases. Therefore, local treatment was not performed, and they received palliative chemotherapy only. None of them lived longer than 2 years.

In the past few decades, there has been a constant effort to improve the chemotherapy regimen and outcome of patients with EWS [13,33,34,35,36,37,38,39]. In Europe, the standard first-line chemotherapy during the study period consisted of VIDE induction therapy and consolidation after local treatment with VAI or VAC. In our study, all patients with localized disease underwent multiagent chemotherapy according to the mentioned standard. HDCT is indicated in all patients with metastatic disease. However, in our cohort, only one of the three patients received HDCT. Two patients had progression under VIDE treatment and were not fit for further treatment.

Our study presents several limitations inherent to the retrospective nature of data analysis and particularly regarding the relatively small sample size. Due to this, our results should be interpreted with caution as they might not be applicable to a broader patient population.

Nonetheless, this paper is the first to describe the prevalence and clinical outcome of primary mediastinal EWS, in a cohort of patients who were included in two large, prospective, randomized trials. Furthermore, all patients were treated according to a homogenous treatment regimen. Despite the limitations, this study gives new insights into the management of this extremely rare disease.

## 5. Conclusions

This is the first study to describe the prevalence and outcome in patients with primary mediastinal EWS treated within two large, international, prospective, randomized trials. Primary mediastinal EWS is extremely rare, with a prevalence of 0.3% among all newly diagnosed EWS patients. Five-year survival was 64%, which is favorable compared to historical cohorts of patients with primary mediastinal sarcoma of all histologies and in line with EWS of different origins. R0 resection can be achieved in a high number of patients if they are treated in specialized centers. Apart from one patient who was lost to follow-up, all patients who had undergone surgery were alive at the end of follow-up. The mediastinal tumor site, even though surgery can be extremely challenging, does not seem to influence the outcome.

Patients with metastases at the time of diagnosis have significantly worse OS compared to those with localized disease. A discussion by a dedicated multidisciplinary tumor board is imperative, in order to offer the best treatment strategy for each patient with this rare disease.

## Figures and Tables

**Table 1:** Characteristics of patients with primary mediastinal ES.

Patient N°	Year of Diagnosis	Study	Age	Tumor Size (cm)	Mediastinal Compartment	Met at Diagnosis	Initial Therapy	Surgery	Rtx (Gy)	OS (Years)
1	2006	EE99	30	11	Middle	-	VIDE + RTX + VAI	-	54	7.0
2	2000	EE99	27	12	Anterior	OSS, HEP	VIDE	-	-	0.8
3	2007	EE99	11	6	Anterior	-	Surgery + VIDE + VAI + RTX	VATS tumor resection (R1)	30	9.6+
4	2001	EE99	49	NA	NA	OSS, PLE	VIDE + VAI + High Dose CHT	-	-	1.8
5	2002	EE99	34	16	Posterior	-	VIDE + Surgery + VAI	En bloc resection incl. intrapericardial lower bilobectomy, pericardium and oesophageal muscle layer (R0)	-	6.1+
6	2002	EE99	21	13	Middle	-	VIDE + Surgery + VAI + RTX	En bloc resection, atrial reconstruction (R0)	45	5.6
7	2009	EE99	4	13	NA	-	VIDE + VAI + Surgery	En bloc resection incl. pericardium, diaphragm and lung wedge (R0)	-	1.4+
8	2008	EE99	31	NA	Anterior	PUL, PER, PLE	VIDE	-	-	0.6
9	2010	E08	46	NA	NA	-	VIDE + VAC + RTX	-	NA	7.6+

OSS = bone, HEP = liver, PLE = pleura, PUL = lung, PER = pericardium, CHT = chemotherapy, RTX = radiotherapy, VATS = video-assisted thoracic surgery, OS = overall survival, R = completeness of resection, EE99 = EUROEWING99, E08 = EWING2008+ = still alive at the time of last follow-up.

## Data Availability

The raw data supporting the conclusions of this article will be made available by the authors on request.
